# Supplementary material for: A solution to the learning dilemma for recurrent networks of spiking neurons
Source: Nat Commun. 2020 Jul 17;11:3625. doi: 10.1038/s41467-020-17236-y (PMC7367848; doi:10.1038/s41467-020-17236-y)
Supplement: Supplementary file 3 — Description of Additional Supplementary Files [file 41467_2020_17236_MOESM3_ESM.pdf]

Description of Additional Supplementary Files

File name: Supplementary Movie 1

Description: Task from Figure 3 with difficult temporal credit assignment. Rodent task from Morcos et al. 2016 [23] and Engelhard et al. 2019 [14] that requires long-term credit assignment for learning: a rodent has to learn to run along a linear track in a virtual environment, where it encounters several cues on the left and the right side along the way. It then has to run through a corridor without cues (giving rise to delays of varying lengths). At the end of the corridor, the rodent has to turn to either the left or the right side of a T-junction, depending on which side exhibited more cues along the way.

File name: Supplementary Movie 2

Description: Dynamics of e-prop for the task from Figure 3 with difficult temporal credit assignment. The computation of the LSNN is accompanied by the computation of synapse specific eligibility traces. An error in the computation only becomes apparent during the so-called decision period at the end of a trial. In this last phase, a learning signal (L_j) that transmits deficiencies of the network output is provided separately to each neuron. As can be seen from the video, synapses that project to neurons with adapting thresholds (ALIF neurons) still have non-vanishing eligibility traces during the last phase ("highways into the future"), and hence can be combined with the learning signals at that time to implement long-term credit assignment.

File name: Supplementary Movie 3

Description: Dynamics of BPTT for the task from Figure 3 with difficult temporal credit assignment. Dynamics of BPTT for the task where temporal credit assignment is difficult: First, a simulation of the network has to be carried out in order to produce the network state of all neurons for all time steps. After that the loss function E can be evaluated. Then the simulated network activity is replayed backwards in time to assign credit to particular spikes that occurred before the loss function became non-zero. One sees that the slow time constants that are present in the dynamics of adapting thresholds of adaptive LIF (ALIF) neurons result in slowly decaying non-vanishing gradients during the backpropagation through time ("highways into the past"). In contrast, for LIF neurons the backpropagated gradients vanish rather quickly.

File name: Supplementary Movie 4

Description: Network dynamics, synaptic plasticity, and performance of an LSNN trained by reward-based e-prop to win the Atari game Pong. A trial of the Atari Pong task after training the network with reward-based symmetric e-prop: The video frames of the game screen (right) are preprocessed using a spiking CNN and provided as input to the LSNN, resulting in spiking activity (bottom left: LIF, ALIF). Output neurons predict future rewards, and the probability of taking actions (Move up, Move down, Stay), shown top left. Learning dynamics are induced when reward prediction errors (green) are combined with a decaying product of local eligibility traces and action feedback (blue). This results in weight changes at synapses (red), shown middle left.
